# A new tool for long-term studies of POM-bacteria interactions: overcoming the century-old Bottle Effect

**DOI:** 10.1038/srep14706

**Published:** 2015-10-05

**Authors:** Danny Ionescu, Mina Bizic-Ionescu, Arzhang Khalili, Reza Malekmohammadi, Mohammad Reza Morad, Dirk de Beer, Hans-Peter Grossart

**Affiliations:** 1 Leibniz Institute for Freshwater Ecology and Inland Fisheries (IGB), Alte Fischerhuette 2, OT Neuglobsow, 16775, Stechlin, Germany; 2 Max Planck Institute for Marine Microbiology, Celsiusstrasse 1, 28359, Bremen, Germany; 3 Sharif University of Technology, Azadi str’, 1458889694, Teheran, Iran; 4 Institute for Biochemistry and Biology, Potsdam University, Maulbeerallee 2, 14469,10 Potsdam, Germany

## Abstract

Downward fluxes of particulate organic matter (POM) are the major process for sequestering atmospheric CO_2_ into aquatic sediments for thousands of years. Budget calculations of the biological carbon pump are heavily based on the ratio between carbon export (sedimentation) and remineralization (release to the atmosphere). Current methodologies determine microbial dynamics on POM using closed vessels, which are strongly biased towards heterotrophy due to rapidly changing water chemistry (Bottle Effect). We developed a flow-through rolling tank for long term studies that continuously maintains POM at near *in-situ* conditions. There, bacterial communities resembled *in-situ* communities and greatly differed from those in the closed systems. The active particle-associated community in the flow-through system was stable for days, contrary to hours previously reported for closed incubations. In contrast to enhanced respiration rates, the decrease in photosynthetic rates on particles throughout the incubation was much slower in our system than in traditional ones. These results call for reevaluating experimentally-derived carbon fluxes estimated using traditional methods.

Particulate organic matter (POM) spans over 6 orders of magnitude in size (from <1 μm to >10 cm) and over 8 orders of magnitude in abundance (<1 L^−1^ to >10^8^ L^−1^)^1^,^2^. POM aggregates consist of homogenous or heterogeneous assemblages of live or senescent organisms^1^.

POM flow to the deeper parts of water bodies forms a critical link in the global carbon cycle called the “Biological Carbon Pump”^3^,^4^,^5^. It is estimated that sinking particles export up to 25% of the carbon captured by phytoplankton in the surface ocean^6^ to deeper water layers. Ca. 20% of this export (~5% of surface values) is buried in the ocean sediments^7^ mainly due to their mineral ballast^8^. During the sinking process, these organic particles are hotspots of microbial activity and represent important loci for organic matter mineralization and nutrient redistribution in the water column^1^,^3^,^9^. The concentration of carbon and nutrients in organic particles can exceed those in seawater by >2 orders of magnitude^10^. Accordingly, this substrate availability leads to particle-associated (PA) bacteria being more active than the free-living (FL) bacteria^11^,^12^,^13^, frequently contributing to >50% of total prokaryotic activity despite their often lower abundance^14^,^15^,^16^,^17^,^18^. Although POM fluxes are frequently determined with sediment traps^19^,^20^, there is a large discrepancy between the POM reaching the bottom and the modeled fluxes based on carbon transport from the euphotic zone^21^,^22^. This suggests that microbial mineralization during particle sinking is still not well understood.

Whether CO_2_ from remineralized POM resurfaces close to where it was formed or at great distances due to oceanic currents is a function of the remineralization depth^23^. Kwon *et al.*^23^ suggested that a change of several meters (20 m) in remineralization depth has a drastic effect on exchange with atmospheric CO_2_. Remineralization depth is dependent on particle ballast, size and shape, all factors that are directly affected by microbial activity^24^. Most global POM flux models ignore particle size and shape^24^,^25^, or deduce the particle sizes from remote sensing data^24^. In either case, mineralization depth is an unknown factor which is usually fitted to match ocean phosphorus distribution^24^. The latter is typically a limiting nutrient and is also affected by microbial activity. Overall current POM flux calculations rely to a certain extent on questionable assumptions, and there is a need for more precise measurements of *in situ* particle degradation rates and temporal changes in particle structure.

Due to the ephemeral nature of particles, their structural heterogeneity and their varying abundance, it is impossible to obtain precise temporal information on the formation and degradation of particles as well as on the successive bacterial colonization and activity responsible for these processes. Therefore, incubation experiments using rolling tanks that allow for the production of large numbers of similar, robust, newly formed macro-aggregates from natural material are the most common experimental approach in controlled POM dynamics studies (e.g.^26^,^27^,^28^,^29^). However, only a few studies using such an approach have followed the temporal change in PA bacterial community composition (BCC)^26^,^27^,^30^,^31^,^32^, and even a fewer studies have tried to link bacterial community composition and its activity on the particles^27^,^33^,^34^, but not in a sufficiently high temporal resolution to resolve the specific microbial processes^33^,^34^.

The above mentioned closed systems suffer from a major drawback by being completely isolated from the natural environment. In such systems there is no continuous supply of bacteria or fresh OM and nutrients, and at the same time toxic metabolites may accumulate within the tank. This creates the “**Bottle Effect**”, a phenomenon that has been known for nearly a century^35^, in which the natural microbial community is rapidly replaced by opportunistic bacteria^35^,^36^,^37^,^38^, alongside an increase in heterotrophic over autotrophic bacteria^39^. These changes have profound implications on the microbial activities on the particles, often leading to overestimation of microbial process rates compared to *in-situ* data^37^,^40^, and even confusing and contradictory conclusions as to whether the oligotrophic ocean is overall heterotrophic^41^ or autotrophic^42^.

To study the long-term POM dynamics without the bottle effect, we have designed and tested a flow-through rolling tank device. This device allows for a continuous flow of water while maintaining the POM aggregates inside in a stable laminar flow-field, thereby allowing us to directly follow the temporal shifts in the diversity and activity of microbial communities on a fine scale under nearly *in-situ* conditions. We compared the flow-through rolling tank system directly connected to Lake Stechlin (northeastern Germany) with a standard closed system. Both systems were inoculated with aggregates formed from an axenic diatom culture. Water samples were collected for community analysis over a period of several days. Aggregates and the surrounding water were collected over time for measurements of bacterial community composition, respiration and photosynthesis. The results showed that the microbial community in the closed tank rapidly changed, but that in the flow-through system remained similar to the *in situ* community for at least 9 days.

## Materials and Methods

### Flow-through rolling tanks

Two different flow-through rolling tanks with different internal water flow-fields were designed and constructed. Additionally, each rolling tank design allows for different types of long-term experiments on particulate matter. The fluid motion in both rolling tanks is controlled by gravity, through-flow and rotation. By combining the associated parameters a variety of flow types can be easily mimicked.

**Figure 1:**
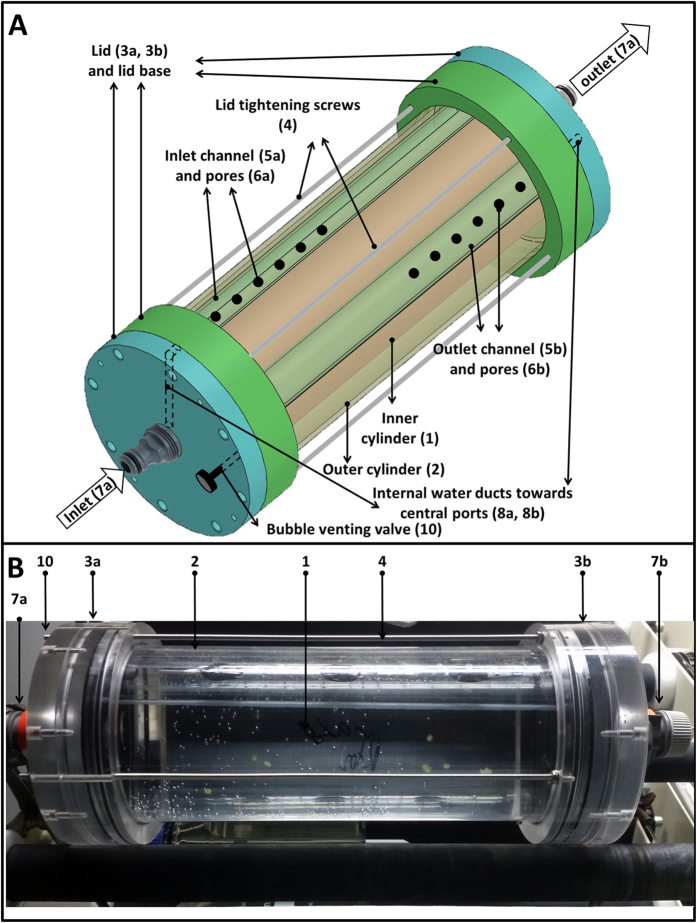
Schematics of the first flow-through rolling tank designed (**A**) and a picture of the system during a running experiment (**B**). The system described here is 30 cm long and the diameter of the inner tube is 9 cm. The inlet and outlet ports are fitter with standard Garden-hose adaptors to allow for the continuous rotation of the device alongside a continuous flow of water. This design consists of a transparent end-sealed inner cylinder (1) in which the particles are placed or allowed to form. A similar outer cylinder (2) encases the inner one, both fit hermetically into the side lids (3a, 3b), which are held fixed together by long fitting screws (4). Water flows into and out of the inner cylinder through two parallel channels 90° apart in clockwise direction (5a, 5b), each with 6 holes. The inlet and outlet holes (6a, 6b) are located on the first and second half of the inner cylinder, respectively. The entrance and exit of water (7a, 7b) lies at the center of the side lids, respectively. From there, water is led into and out of the corresponding inlet and outlet channels via inner tubes (8a, 8b). By connecting the entrance and exit of water with standard garden-hose swivel tube-connectors (9a, 9b), the entire system can rotate independently of the external pipes. A bubble-venting port (10) is also mounted on one of the side lids. Detailed images of the individual parts and their dimensions are given in Fig. S1 and Table S1, respectively.

**The first design** (Figs 1, S1) consists of a transparent end-sealed inner cylinder (1) in which the particles are placed or allowed to form. A similar outer cylinder (2) encases the inner one, both fit hermetically into the side lids (3a, 3b), which are held fixed together by long fitting screws (4). Water flows into and out of the inner cylinder through two parallel channels 90° apart in clockwise direction (5a, 5b), each with 6 holes. The inlet and outlet holes (6a, 6b) are located on the first and second half of the inner cylinder, respectively. The rationale for this arrangement is to prevent direct flow of water between the two channels as well as to assure the proper exchange of water within the inner cylinder. The entrance and exit of water (7a, 7b) lies at the center of the side lids, respectively. From there, water is led into and out of the corresponding inlet and outlet channels via inner tubes (8a, 8b). By connecting the entrance and exit of water with standard garden-hose swivel tube-connectors (9a, 9b), the entire system can rotate independently of the external pipes. A bubble-venting port (10) is also mounted on one of the side lids. Detailed images of the individual parts and their dimensions are given in Fig. S1 and Table S1, respectively. This design is easy to sample and therefore suitable for performing sacrificial experiments.

**Figure 2:**
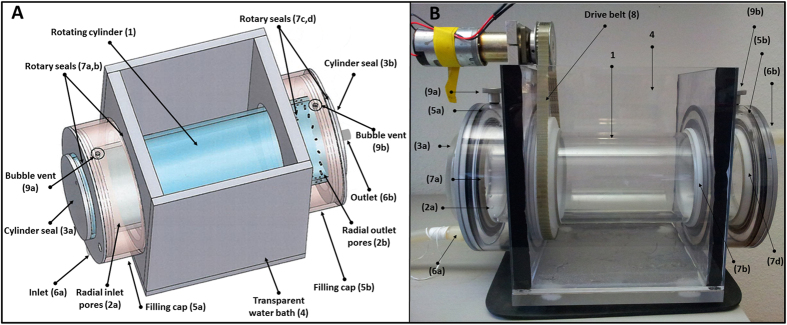
Schematics of the second flow-through rolling tank designed (**A**) and a picture showing the mounted motor and rotating belt (*B*). The system described here is 30 cm long and the diameter of the inner tube is 9 cm. This design consists of a single cylinder (1) with 20 peripheral holes (2a–b) at each side through which water flows into and out of the cylinder. The cylinder is end-sealed with removable caps (3a–b). A transparent rectangular container mounts the cylinder (4). Two cylindrical caps (5a–b) encapsulate the inlet and outlet holes and act as reservoirs for entering and exiting water (6a–b) into and out of the cylinder. Both caps and the rectangular mount are stationary while the cylinder is mounted with rotary seals (7a–d) and may rotate by drive-belt (8). Bubble venting valves (9a–b) are mounted on both fixed caps. Detailed images of the individual parts and their dimensions are given in Fig. S2 and Table S2, respectively.

**The second design** (Figs 2, S2) consists of a single cylinder (1) with 20 peripheral holes (2a–b) at each side through which water flows into and out of the cylinder. The cylinder is end-sealed with removable caps (3a–b). A transparent rectangular container mounts the cylinder (4). Two cylindrical caps (5a–b) encapsulate the inlet and outlet holes and act as reservoirs for entering and exiting water (6a–b) into and out of the cylinder. Both caps and the rectangular mount are stationary while the cylinder is mounted with rotary seals (7a–d) and may rotate by drive-belt (8). Bubble venting valves (9a–b) are mounted on both fixed caps. If particle tracking is desired, the rectangular mount can be filled with water to eliminate the optical distortion caused by the curvature of the cylinder. This can be further used as a water bath to regulate the temperature inside the rolling tank. Detailed images of the individual parts and their dimensions are given in Fig. S2 and Table S2, respectively.

### Algal culture

An axenic culture of a freshwater diatom (*Navicula* sp.) isolated from Lake Stechlin, was grown in 900 mL of sterile Z-medium^43^ in 1 L Shott Duran bottles. The culture was grown under a 12 h dark-light cycle at 15 °C for 2 weeks. Afterwards, the diatoms in the bottles were placed on a roller table to promote aggregation of diatom cells prior to inoculation with fresh water from Lake Stechlin in the flow-through system.

### Testing for the “Bottle Effect”

One closed and one flow-through systems (first design) were compared to test for the “bottle effect”. Freshly formed diatom aggregates were carefully transferred to the water-prefilled inner cylinder of the rolling tank avoiding air bubbles. The device was then sealed and allowed to roll for up to an hour before initiating lake water flow. A sieve with a mesh size of 100 μm was used to prevent larger particles from entering the device. The sieve was checked daily and cleaned to avoid accumulation of objects and formation of biofilms. In the bottle effect tests the rotational speed of the closed and the flow-through systems was set to 2 rpm. The flow rate for the flow-through system amounted to 5 ml min^−1^. The closed system was inoculated in the same manner after which no further natural water was allowed to enter.

A volume of 50 ml water was sampled daily from the closed system for 4 days and from the inlet and outlet of flow-through rolling tank on the 1^st^, 5^th^ and 9^th^ day. In parallel, water was also sampled directly from Lake Stechlin. Throughout the experiment, there was a continuous flow of water from Lake Stechlin into the flow-through rolling tank. The sampled water was filtered through a polycarbonate filter with a pore size of 0.22 μm (47 mm Ø; Sartorius, Germany) and DNA was extracted as described below.

### Comparing microbial activities in closed vs. flow-through rolling tanks

Individual aggregates (n = 3–4) were sampled from the flow-through rolling tank daily for 5 days and once more 9 days after inoculation. Aggregates from the closed system (n = 4) were collected only 5 days after inoculation to avoid opening and closing the system and thus changing the inner conditions. Though the closed system was inoculated with an equal number of diatom particles as the open system, by the time of sampling they coagulated into 4 large aggregates. Upon collection, aggregates were transferred to a net-jet measurement chamber, submersed in fresh lake water, for microsensor analysis as described elsewhere^44^.

### Gross photosynthesis measurements

Oxygen measurements were conducted with a Clark-type microelectrode with a guard cathode^45^. Gross photosynthesis was measured at the O_2_ peak using the light dark shift method^46^. Following the measurements individual aggregates were fixed for 1h in a 1% formaldehyde solution at room temperature (~22 °C) after which they were placed on polycarbonate filter, dried by vacuum and stored at −20 °C until further analysis.

### Chlorophyll *a* measurement by hyperspectral analysis

The chlorophyll *a* content of each aggregate which was measured with microsensors, was analyzed under the microscope (Zeiss Axiovision; magnification 200X) using a hyperspectral camera (PIKA II, Resonon inc.) as described in Ionescu *et al.*, 2012^47^. Chlorophyll *a* content for each pixel of the hypercube image was determined according to Chennu *et al.*, 2013 using the HyPurveyor software^48^.

### Temporal changes in particle-associated communities in the flow-through system

To observe temporal changes on particle-associated bacterial communities, 6 rolling tanks were inoculated and sampled in pairs after 1, 7 and 8 days. Each rolling tank was opened and single macroscopic aggregates (SMA) were carefully collected with a cut-end syringe while trying to avoid collection of ambient water. Collected SMA were rapidly gathered and filtered through a polycarbonate filter of 5 μm pore size (47 mm Ø; Sartorius, Germany) to remove free-living bacteria.

### DNA and RNA extraction

After filtration, filters for RNA analysis were immediately placed in Z6-buffer (8 M guanidinium-HCl, 20 mM MES, 20 mM EDTA [pH 7.0] and 0.7% [v/v] 2-mercaptoethanol) to deactivate RNAses and denature all proteins. The samples were then stored at −80 °C till further processing.

For transcriptomics analysis (data to be presented elsewhere) we have enriched the mRNA proportional concentration by removing rRNA transcripts following Steward *et al.*^49^. First strand cDNA was created using the Superscript III kit (Invitrogen) according to the manufacturer’s instructions.

DNA and RNA were extracted using the hot phenol method^50^ and sequencing was done at MR. DNA (Molecular Research LP), Shallowater, TX). Sequences were deposited in the European Nucleotide Archive (ENA) under study number PRJEB7963 (http://www.ebi.ac.uk/ena/data/view/PRJEB7963). cDNA samples from duplicate rolling-tanks were combined prior to sequencing. Three metatranscripome data sets generated in this study are available in MG-RAST as public databases under numbers: 4552440-4552442.

Microbial community composition from the transcriptomics data was reconstructed using EMIRGE^51^ using the default parameters and the SILVA SSU (V. 111)^52^ database. The obtained OTU abundance was used to create artificial fasta files containing 10,000 sequences. Bacterial community analysis of all experiments was done using the SILVA NGS pipeline as previously described^47^.

### Governing equations for modeling of fluid flow and particle dynamics

To demonstrate the feasibility of the flow-through devices suggested here, a mathematical model has been implemented to explore the terms and conditions of the particle movement within both types of devices. The governing equation of motion in a rotating coordinate frame is the incompressible Navier-Stokes equation given by


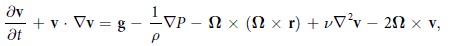


in which **v**, *t*, *P*, *ρ*, **g**, *v* and **Ω** denote velocity vector, time, fluid pressure, fluid density, vector of acceleration due to the gravity, kinematic viscosity and angular velocity vector, respectively. Furthermore, the symbol 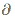 stands for partial derivative while the symbol 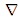 is the gradient operator for differentiation with respect to space coordinates. The coordinates of the velocity vector are *u*, *v*, *w*, in xyz direction, respectively. The rolling tanks undergo a rotation with the angular velocity vector of **Ω** = (0, 0, *ω*) with *ω* as rotations per minute (rpm).

The above flow equation has been solved by COMSOL Multiphysics 4.3 in a Cartesian coordinate system (xy*z*) with x in the lateral direction, y in the direction opposite to the gravity and z being aligned with the central axis. From the steady state solution of the velocity and the pressure field we calculate the drag forces acting on each solid particle. Newtonian dynamics has been employed to calculate the particle’s acceleration, velocity and path as a function of time and space. The forces acting on each particle are the drag and the body force due to gravity. The first one has been obtained from the solution of the Navier-Stokes equation. This is included in the particle tracing module of COMSOL. The no-slip boundary conditions have been employed at all solid walls. The boundary condition at the inlet and outlet holes is given by setting a constant flow rate. Initially, we used zero velocity and atmospheric pressure everywhere in the computational domain. The stability of the solutions has been ensured by a grid refinement procedure for finding the minimum necessary cell volumes.

## Results

### Fluid flow and particle dynamics

There have been several analyses published on the experimental conditions in the rotating cylinders undergoing a solid body rotation^53^,^54^,^55^. The flow field and the particle dynamics have been investigated in this work computationally in both flow-through designs and are compared with the results of a closed system. Throughout the computations, the flow rate and the rotational speed have been set to the constant values of *Q* = 9 ml/min and *ω*_*z*_ = 1.8 rpm, respectively.

The computation of the fluid flow has been shown by the streamlines of a massless particle, with a density equivalent to that of the ambient water (ρ = 1000 kg m^−3^) with the usual value of 1.0 × 10^−6^ m^2^ s^−1^ as the kinematic viscosity. To describe the particle dynamics we follow the motion of a spherical particle (i.e. pathline) of 1 mm diameter with a density equal to that of the aggregate (ρ = 1005 kg m^−3^)^8^.

**Figure 3:**
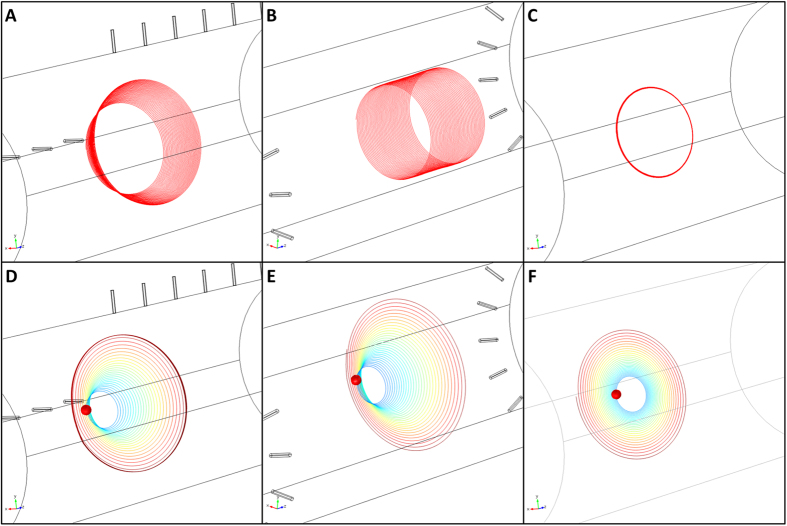
First row: 3D streamlines of massless particles released at (x, y, z) = (2 cm, 0, 13.5 cm) in the first flow-through design (3a), second flow-through design (3b) and closed system (3c). Second row: 3D pathlines of the a spherical particle with a density of ρ = 1005 kg m^−3^ 8 released from the same position in the first design (3d), second design (3e) and the closed system (3f). The red circle denotes the initial position of the particle at t = 0 and its path after 1000 seconds.

In Fig. 3, the streamlines for a massless particle (first row) and the pathlines of the particle under study (second row) have been shown for the first design (first column), second design (second column) and the closed rotating system (third column). In all images, the initial particle position is given by (*x*, *y*, *z*) = (2 cm, 0, 13.5 cm). The massless particle released from this position in the first design, rotates on paths with increasing radius accompanied with a simultaneous flow component in the *z* direction (Fig. 3a), while the same particle in the second design moves on spiral path with a constant distance to the central axis (Fig. 3b). Note that the difference between the streamline patterns of the first and second design is associated with the different way the inlet and outlet ports are constructed. In contrast to these two flow-through designs, the particle path in the closed system resembles the rotation of a solid body without an axial component (Fig. 3c) and are similar to those obtained by Jackson^53^ as well as Engel and Shartau^55^.

The second row of Fig. 3 is devoted to the computation of the pathlines of the particle (with initial position denoted by the red circle) in the first design, second design and the closed system, respectively. With the given combination of *Q* and *ω*_*z*_, aggregates released at the mid-*z*-plane (*z* = *13.5* cm) display an almost equivalent path (Fig. 3d,e) with slight differences, not visible by naked eyes, moving along the *z* direction. In contrary to flow-through designs, the particle path in the closed system (similar to those obtained by Jackson^53^ as well as Engel and Shartau^55^) follows the solid body rotation and the particle approaches the wall via a circular pathway. The elapsed time for all these images is 1000 seconds.

As far as the coagulation dynamics is concerned, different flow dynamics trigger different coagulation scenarios resulting in different aggregate sizes and properties. Jackson^53^ demonstrated the role of constant container rotation on the nature of the coagulation in a closed system. Obviously, the coagulation dynamics in the presence of a through-flow as given in our devices will differ from those in closed systems. However, this issue will not be addressed here.

**Figure 4:**
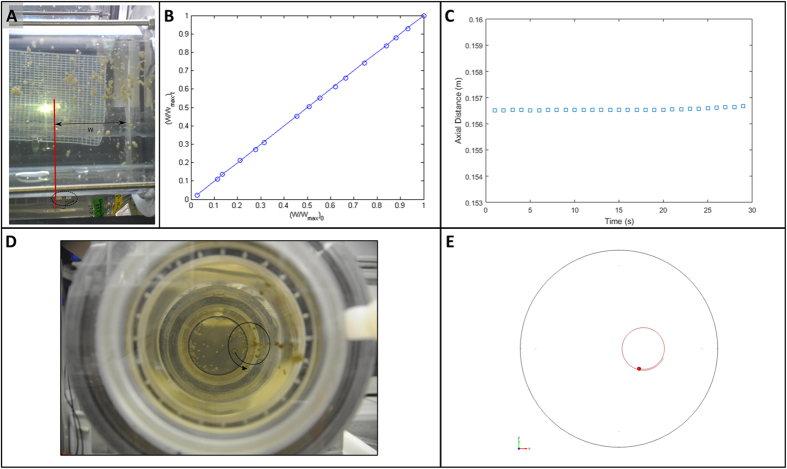
(**A**) We draw a red line on the left edge of the white band glued on the outer cylinder, highlighted by the black ellipse. From this fixed reference line, the horizontal displacement of each particle from its initial position can be traced versus time. (**B**) The experimentally captured relative displacements (W/W_max_)_t_ of 15 randomly chosen particles from 30 subsequent images have been plotted versus their initial displacements (W7W_max_)_0_. All particles were found to move in the axial direction from their original positions only by less than 0.8% hinting toward a quasi-2D spiral motion. (**C**) Numerically obtained axial displacement the aggregate particles versus time. (**D**) The experimentally determined path of a randomly selected particle after 50 seconds. (**E**) The counterpart of image d as predicted by numerical simulation.

To validate the numerical results, we performed experiments using 3D video photography with both designs with the same flow rate and rotational speed. Two examples are investigated here. In the first example, we compare the particle motion in the *y,z*-plane for a series of 30 images within 30 seconds with the same result obtained numerically in the first design (Fig. 4a–c). A reference line (the vertical red line in Fig. 4a) has been drawn which is fixed to a point on the cylinder periphery (the left edge of the white band glued on the outer cylinder, highlighted by the black ellipse). With the focus on 15 randomly selected particles, each being distant away from the reference line by W_i_ (i = 1,2, …, 15), we recognized from the successive images that W_i_ remained almost constant throughout the visualization time of 30 seconds. A further careful analysis of the particle displacements was obtained by scaling W_i_ for all particles with the maximum displacement W_max_ and plotting the initial versus temporal displacements (Fig. 4b). The maximum standard deviation from the perfect stationary state is within 0.8% hinting toward a negligible axial velocity component of all particles within the time period mentioned. This confirms the finding of the numerical simulation shown in Fig. 4c.

In the second example, the path of a randomly chosen particle in the *x,y*-plane has been captured from a series of 30 images within 50 seconds (Fig. 4d). Evidently there is a good qualitative agreement between the spiral path obtained from the experimental visualization and that of the corresponding numerical prediction within the same time period (Fig. 4e). As can be verified, there exists a good agreement between the numerical and the experimental results.

### Quantification of the “Bottle Effect”

**Figure 5:**
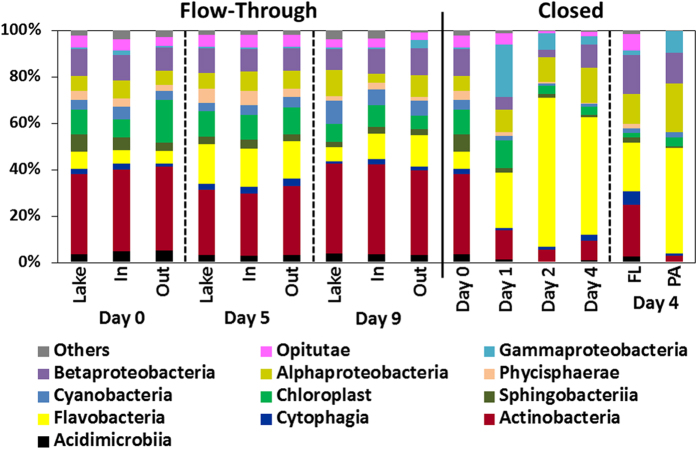
Bacterial community composition at the class level of the in the flow-through and closed rolling tank system. The community in the flow-through rolling tank was sampled at the outlet of the tank and is presented together with the community *in-situ* in the lake as well as at the inlet to the tank following the pumping of water. In addition to total community analysis (>0.2 μm), on the fourth day, the free-living (FL; >0.2 μm & <5 μm) and particle-associated (PA; >5 μm) communities were analyzed separately.

The first question is whether or not the particles in the flow-through rolling tank were exposed to a similar bacterial community as they were in the natural environment. To answer this, we examined the total microbial community (FL and PA) at the rolling tank outlet vs. the one in the Lake Stechlin. The second question is if the pumping procedure or equipment had an effect on the bacterial community (e.g. due to clogging or biofilm formation). For this purpose, we monitored the microbial community at the rolling tank inlet (Fig. 5). These results were compared to the bacterial community developing in a standard closed rolling tank inoculated on the same day (Fig. 5). The closed system demonstrated a strong bottle effect with the bacterial community changing in <24 h. These changes occurred in both the FL and PA fractions (Fig. 5). While initially the single most abundant group in the closed system were *Actinobacteria*, after 24 h the community was dominated by *Flavobacteria*, an effect that grew stronger in the following days and was noticeable both in the FL and the particle-associated communities (Fig. 5). In contrast to the closed system, the community samples at the inlet and outlet of the flow-through rolling tank were nearly the same as those sampled in the lake for the entire duration of the experiment indicating the absence of methodological biases, e.g. due to biofilm formation inside the tubes etc.

**Figure 6:**
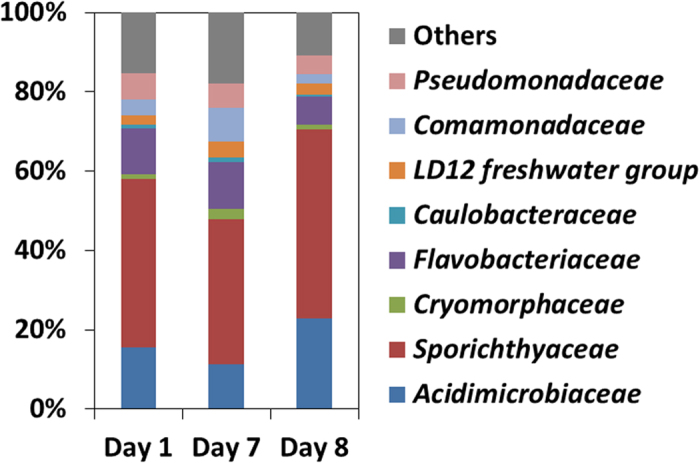
Bacterial community composition at the family level as obtained from RNA sequences of pooled single macroscopic aggregates from flow-through rolling tanks sampled after 1, 7 and 8 days from inoculation. Each sample is a result of 2 combined rolling tanks.

The similarity and stability of the total bacterial community at the inlet and outlet of the flow-through rolling tank show that no major changes occurred during the experiment neither in the FL nor in the PA communities. Surprisingly, the active PA bacterial community as indicated by the RNA sequences did not change throughout the experiment (Fig. 6) and was found to be dominated by the *Sporichthyaceae* (*Actinobacteria*), *Acidimicrobiaceae* (*Actinobacteria*) and *Flavobacteraceae* (*Bacteroidetes*) families.

### Enhanced particle degradation in the closed system

**Figure 7:**
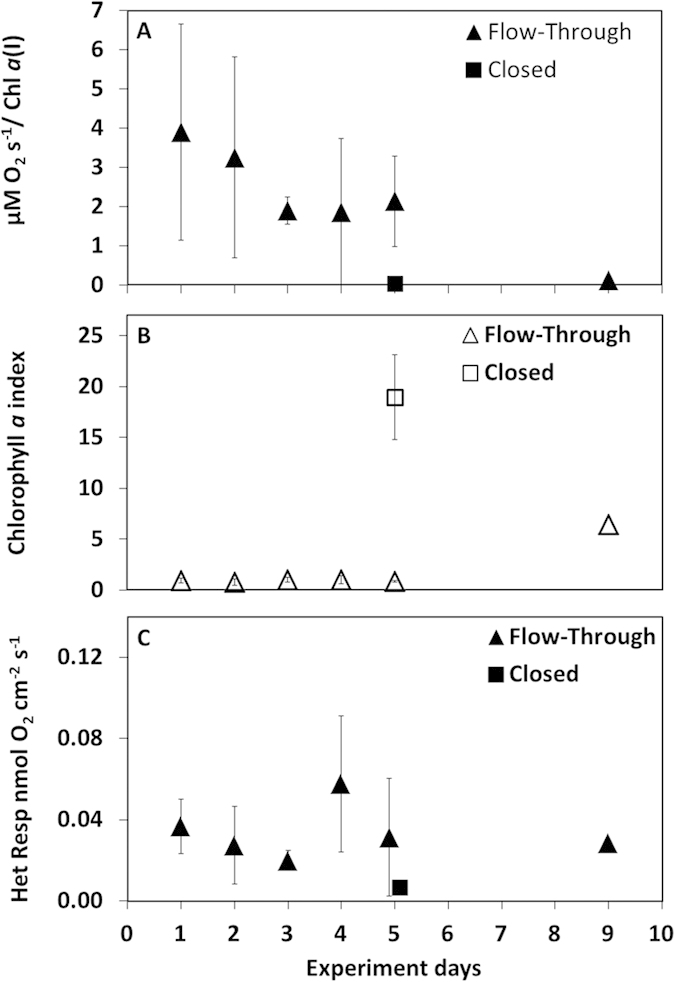
Gross Photosynthesis as measured using the light/dark shift method in individual particles collected from the closed and flow-through rolling tanks (**A**). The Gross Photosynthesis rate was normalized to the Chl *a* index of each particle. The Chl *a* index is a comparable value between particles and was obtained by scanning individual particles with a hyperspectral camera connected to a light-microscope. The average Chl *a* indexes per sampling day are presented as well (**B**). Heterotrophic respiration (**C**) was calculated as the difference between the gross and the net photosynthesis rates for an integrated depth of 100 μm around the O_2_ peak in the particle.

The activity on the diatom particles as evident by a decrease and eventually cessation of photosynthetic activity differed as well between the two systems (Fig. 7A). While in the closed system photosynthesis was undetectable on any particle after 5 days of incubation, gross photosynthesis of aggregated diatoms in the flow-through system (normalized to *Chl a* content) was on average half of the initial values (Fig. 7A). Additional differences were evident in the *Chl a* content of the individual particles (Fig. 7B) with the ones from the closed system containing more *Chl a* even when compared to 9 days old aggregates from the flow-through system. This is despite the fact that aggregates from the flow-through system were exposed to natural water and acquired additional diatoms from the lake. Despite the decrease in photosynthetic activity on the particles, there was no increase in heterotrophic respiration in the flow-through rolling tank.

## Discussion

The flow-through rolling tank devices we present here allow for long term experimentation on any particulate matter at nearly *in-situ* conditions or with controlled settings. While for continuous cultures of suspended cells chemostats have been available for decades and have provided a means to avoid the “Bottle Effect”, no such systems have been so far available for studies using a sufficiently high number of particles.

Our experiments clearly demonstrate that in the flow-through rolling tanks the particles were exposed to the same microbial community as in the natural environment. This is an advantage compared to a closed system in which the microbial community rapidly deviates from its original composition. Subsequently, the overall activity on the particles differed markedly between the flow-through and closed systems. The photosynthesis rate of particles in the closed system decreased to nearly zero within 5 days of incubation. At the same time the chlorophyll content (or degradation products) of these particles was >20 time higher than that of particles in the flow-through rolling tank. This is a direct consequence of enhanced bacterial degradation in closed systems, leading to stickier particles and faster aggregation into large aggregates. At this stage, the difference in particle size and shape was even visible to the naked eye with the particles from the closed system being larger, darker in color and stickier (Fig. S3). Only after 9 days particles incubated in the flow-through system began to show the same characteristics as the 5 days old particles in the closed rolling tank (i.e. color, stickiness, chl *a* index, etc’). This suggests that photosynthetic activity of particles in the euphotic zone remains high for a longer time than has been estimated using closed systems. The increased microbial activity, lack of photosynthesis and exchange with lake water are also evident in the reduced O_2_ concentration measured in the closed rolling tank as compared to the flow-through system (Fig. S4). This suggests that by exposing the particles to nearly *in-situ* conditions we reduced the previously demonstrated bias towards heterotrophy^39^,^40^,^41^. These results are in line with the heterotrophic respiration on the particles on the 5^th^ day of incubation (Fig. 7C). A relatively constant respiration rate on the particles in the flow-through system suggests that a suitable carbon source was constantly available either directly from the still active photosynthesis, from the particle’s EPS, or directly from the water column due to the continuous flow. In contrast, the low respiration rates on the particles from the closed system, together with the evidence of earlier enhanced activity (particle stickiness), suggest that all “labile” primary carbon sources had been exhausted and the residual substrates were harder to degrade or were unsuitable for the PA community. This suggests that while in the photic zone, remineralization of dissolved organic matter that adheres to the aggregate, or originates from photosynthetic exudates, is probably higher than previously estimated. In contrast, the net loss of aggregate-biomass is lower, thus, these less compact particles (Fig. S3A) sink slower and reside longer in the photic zone and are eventually transported intact into the mesopelagic zone.

So far, only a few studies reported temporal changes of particles or their associated microbial communities^26^,^27^,^30^,^31^,^32^, mostly using artificial particles in closed systems. Employing the new flow-through rolling tank we followed the active community attached to individual particles, exposed continuously to the natural community and found it to be stable throughout the whole 8 day-experiment. This finding is in agreement with the much slower decrease in photosynthetic rates and the constant respiration rates on the particles in flow-through rolling tanks comparing to the closed system. We propose that the initial resources provided by the dying particle were never fully consumed due to continuous supply of fresh OM, and thus there was no driving force for the community to exchange. This is in contrast to previous studies suggesting dramatic community shifts already within hours of particle incubation^31^,^56^.

Overall, our results show that the microbial community data obtained by previous studies using closed rolling tanks are not representative of the natural conditions. Moreover, rate measurements of photosynthesis and activities of heterotrophic bacteria on particles might contain large uncertainties.

### Advantages of the flow-through system over the conventional closed rolling tanks

It is important to understand the mechanisms governing POM degradation and transport and their contribution to the macro- and micro-environment throughout the entire water column. These processes cannot be explained adequately with the conventional methodologies, e.g. closed rolling tanks. For example, mineral ballast formed in POM particles is significant to sinking rates and is believed to prevent particle degradation^57^. This, however, is still being studied in closed systems despite the diminishing effects heterotrophic activity may have on carbonate mineral formation due to respiration-based acidification.

The net-jet system introduced by Ploug and Jørgensen^44^ can serve as a nearly *in-situ* platform for measurements on particles and has been used also in this study. However, due to the unique properties of individual particles, it is impossible to keep in suspension more than few particles for any period of time.

The flow-through rolling tanks minimize any unnatural change in bacterial communities due to the bottle effect. They allow continuous exposure of large numbers of organic and inorganic particles in the system to near *in-situ* conditions including not only the natural microbial fauna but also dissolved organic matter, nutrients and a natural community of phages, the latter of which play a major role in the dynamics of FL and PA communities^58^,^59^. Finally, unlike the conventional closed systems, the flow-through rolling tanks enable continuous and simultaneous measurements of solutes, gases, and dissolved and particulate organic matters both at the inlet and the outlet.

### Future applications of the flow-through rolling tanks

Flow-through rolling tanks are integrative tools which can provide accurate numerical values for global carbon models. Simultaneously, they can be used to determine microbial activity on a number of individual particles which in turn can be tracked for long durations to evaluate temporal changes in size and shape. The net release of N, P and DOM due to microbial particle degradation can be continuously monitored and attributed to a known POM mass. Thus, empirical values for the necessary link between global N and P distribution and POM remineralization can be obtained. This will allow validating or correcting current models of POM fluxes using empirical data from a nearly *in-situ* experimental device.

Flow-through systems can be further used to address key ecological questions regarding the dynamics of particulate matter, i.e. elucidating the role of various zoo- and phytoplankton in particle formation and degradation. Since the water is fed into the flow-through systems from a natural source, different mesh sizes can be placed at the inlet to select for certain functional groups and organic matter size fractions. This allows for the study of interactions between particles and different groups of organisms.

To conclude, we described effective flow-through rolling tank devices for long-term studies of organic and inorganic particles while avoiding the bottle effect. The devices are easily operated for routine oceanographic and limnic studies focusing on the ecology, geochemistry, genetics and physics of particulate organic and inorganic matter. Results from long-term experiments at near *in situ* conditions using such devices can be used to augment sediment trap data^21^ and improve our understanding of the global carbon flux.

## Additional Information

**How to cite this article**: Ionescu, D. *et al.* A new tool for long-term studies of POM-bacteria interactions: overcoming the century-old Bottle Effect. *Sci. Rep.* **5**, 14706; doi: 10.1038/srep14706 (2015).

## Supplementary Material

Supplementary Information
