# Supplementary material for: A new tool for long-term studies of POM-bacteria interactions: overcoming the century-old Bottle Effect
Source: Sci Rep. 2015 Oct 5;5:14706. doi: 10.1038/srep14706 (PMC4592964; doi:10.1038/srep14706)
Supplement: Supplementary Information [file srep14706-s1.pdf]

A new tool for long-term studies of POM-bacteria interactions: overcoming the century-old Bottle Effect

Supplementary Material

Ionescu Danny[1,2*], Bizic-Ionescu Mina[1,2], Khalili Arzhang[2], Malekmohammadi Reza[2], Morad Mohammad Reza[3], de Beer Dirk[2], Grossart Hans-Peter[1,4]

[1] Leibniz Institute for Freshwater Ecology and Inland Fisheries (IGB), Altefischerheutte 2, 16775, Neuglobsow, Germany
[2] Max Planck Institute for Marine Microbiology, Celsiusstrasse 1, 28359, Bremen, Germany
[3] Sharif University of Technology, Azadi str', 1458889694, Teheran, Iran
[4] Institute for Biochemistry and Biology, Potsdam University, Maulbeerallee 2, 14469,10 Potsdam, Germany

* Correspondence to: ionescu@igb-berlin.de

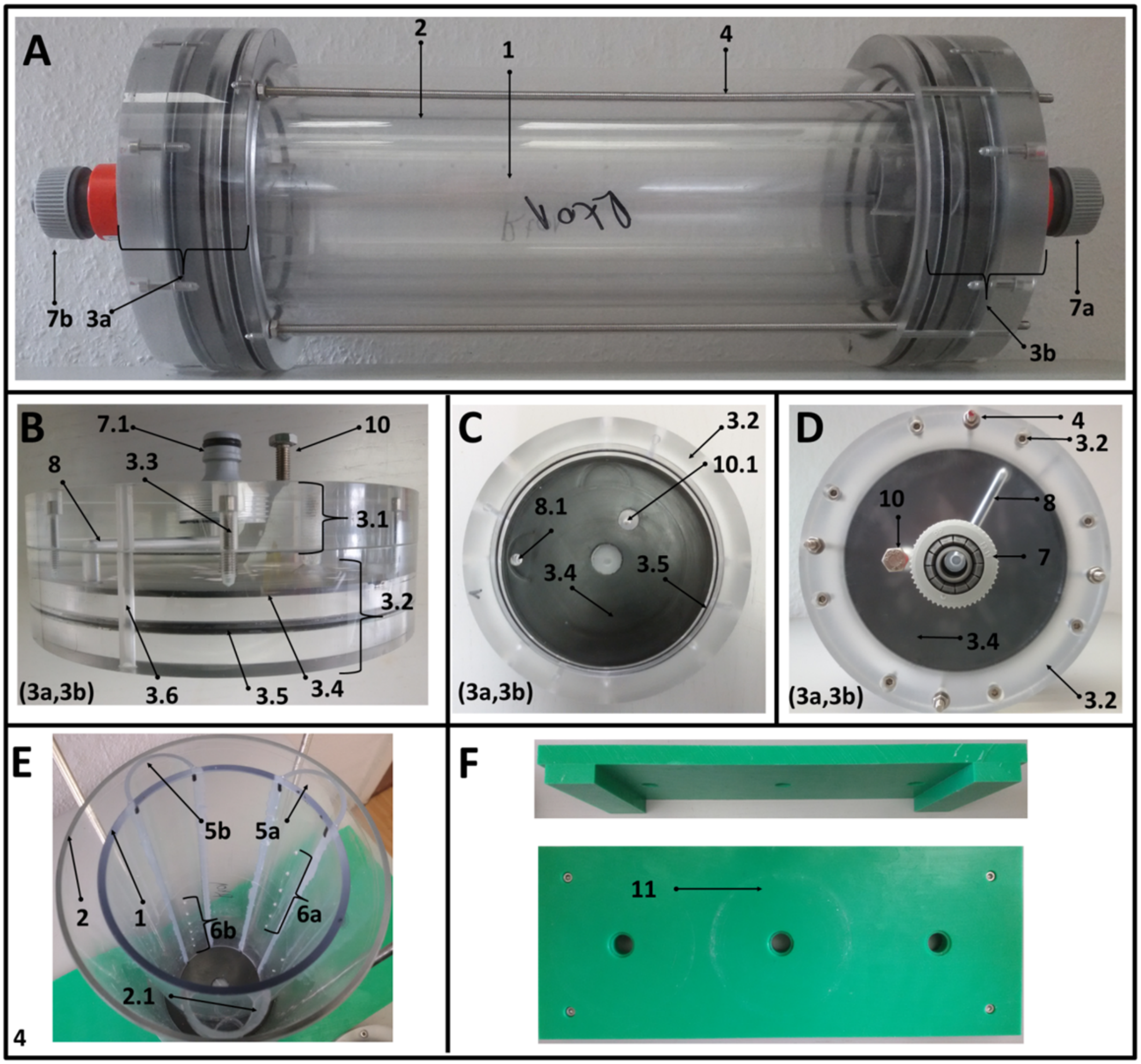

Fig S1 Type-1 flow through rolling tank (A) and its constituting parts: the lids (B-D), the reactor body (E) and a working stand (F). The water flow path is described in the main text. The dimensions of the individual parts are given in Table S1.

Table S1. Descriptions and dimensions of the parts making up the type-1 flow-through rolling tank. Part numbers refer to Fig S1.

Part #	Description	Dimensions (if relevant)	Remarks	Appears in panel:
1	Rolling tank experiment chamber	Ø 90 mm (out)<br>Ø 84 mm (in)<br>300 mm long		A,E
2	Structural case	Ø 120 mm (out)<br>Ø 114 mm (in)<br>300 mm long	Allows fitting of water inlet channels.<br>Can be filled with water for clearer photography.<br>Can be fitted with water ports for temperature control.	A,E
3	Rolling tank lid		The bubble vent is mounted only on part 2a. Holes for fastening screws (3) go through part 2a and are only partial in part 2.	A,B,C,D
3.1	Upper part of lid	Ø 150 mm		A,B,D
3.2	Lower part of lid	Ø 150 mm (out)<br>Ø 120 mm (in)		A,B,C
3.3	Tightening screws			A,B,D
3.4	Rubber seal	Ø 120 mm	Material should not allow bubble accumulation under or within the seal.	B,C,D
3.5	O-ring	Ø 120 mm		A,B,C,D
3.6	Channel for reactor tightening screws (3)	Ø 5 mm	Partial in part 2, through in part 2a	A,B,D
4	Reactor tightening screws	Ø 5 mm<br>330 mm long	Screwed fixed into part 2, tightening (manual screwing) into part 2a	A,D
5	Water inlet channels	Ø 30 mm<br>300 mm long	Half tubes placed 90° one to the other between the casing and the rolling tank experiment chamber	E
6a	Water inlet pores	6 X Ø 0.5 mm	6 ports starting 350 mm from part 2a. Ports are at 200 mm intervals.	E
6b	Water outlet pores	6 X Ø 0.5 mm	6 ports starting 350 mm from part 2. Ports are at 200 mm intervals.	E
7	Hose connector		Standard garden-hose connector (swivel part)	A
7.1	Adapter for hose connector		Standard adapter for garden-hose connector (fixed part)	B
8	Water duct	Ø 5 mm	Tube to divert water from/to central inlet/outlet port to lateral inlet/outlet channels (4.3)	B,D
8.1	Water inlet to lateral channels	Ø 5 mm		C
10	Bubble vent	Ø 5 mm	Vent is capped with a standard stainless steel screw	B
10.1	Inner side of bubble vent	Ø 5 mm		C
11	Working/loading stand	500 X 200 mm	Elevated stand for 3 rolling tanks. Holes are necessary to fit part 2.1	F

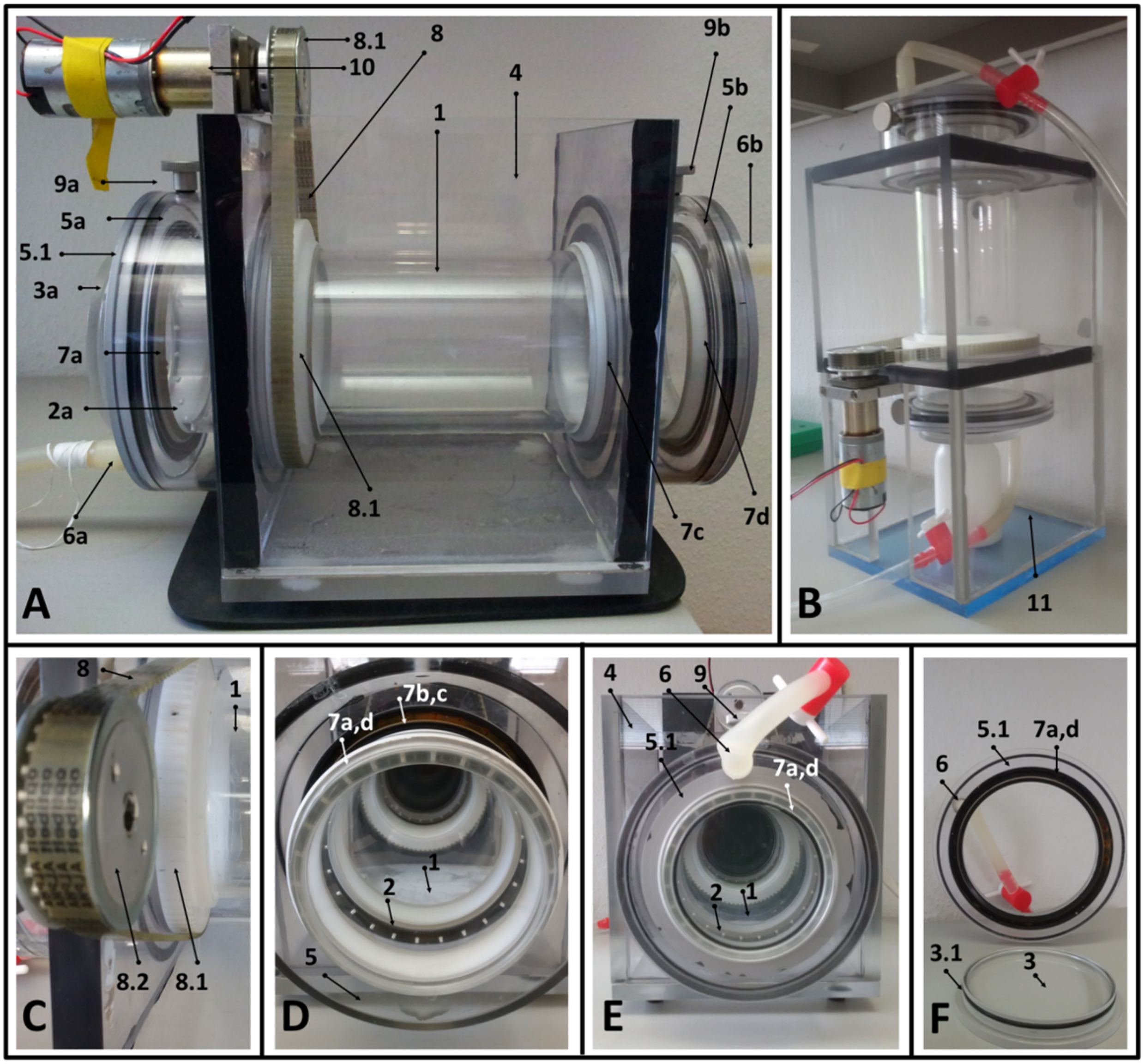

Fig S2 Type-2 flow through rolling tank (A) and its constituting parts: A supporting stand (B), the rotation mechanism (C), the filling chambers (D,F). The water flow path is described in the main text. The dimensions of the individual parts are given in Table S2.

Table S2. Descriptions and dimensions of the parts making up the type-2 flow-through rolling tank. Part numbers refer to Fig S2.

Part #	Description	Dimensions (if relevant)	Remarks	Appears in panel:
1	Rolling tank experiment chamber	Ø 100 mm (out)<br>Ø 90 mm (in)<br>300 mm long		A,D,E
2	Peripheral water inlet holes	20 X Ø 0.5 mm	The holes are evenly spaced on the periphery of each ends of the main reactor 1 cm from the seal	A,D,E
3	Main reactor seal	Ø 100 mm (out)<br>Ø 90 mm (in)<br>15 mm height<br>10 mm penetration depth	The seals closed by pressure alone without locking mechanisms.	A,F
3.1	Sealing O-ring	Ø 90 mm		F
4	Transparent water bath	190x190x200 mm (WxLxH)	The bath walls have a thickness of 10 mm which is included in the overall dimensions.	A,B
5	Filling cap	Ø 150 mm<br>50 mm long	The caps are glues on to the walls of the water bath on one end and sealed with lids on the other.	A,B,D,E
5.1	Filling cap lid	Ø 150 mm out<br>Ø 100 mm in	The lid is fitted with a rotary seal (7a,d)	A,F
6	Water inlet/outlet	Ø 10 mm		A,B,E,F
7	Rotary seals	Ø 103 mm out<br>Ø 100 mm in	Four rotary seals are mounted on the main reactor: 2 at the interface with the water bath (7b,c) and 2 at the interface with the lids of the filling caps (7a,d)	A,D,F
8	Drive belt	100 m wide	Length can be decided based on elevation of motor	A,B,C
8.1	Reactor gear	Ø 100 mm in<br>Ø 110 mm out	Gear mounted and glued around the main reactor	A,B,C
8.2	Motor gear	Ø 50 mm	Size is significant for the maximal rotation speed	A,B,C
9	Bubble vents	Ø 5 mm	Drilled on both filling caps and closed with an O-ring fitted screw.	A,E
10	Motor		We used a 24 V motor connected to an adjustable power supply.	A,B
11	Stand		The white knob in the center of the stands allows pressing the lids in without pushing the main reactor out.	B

Figure S3.

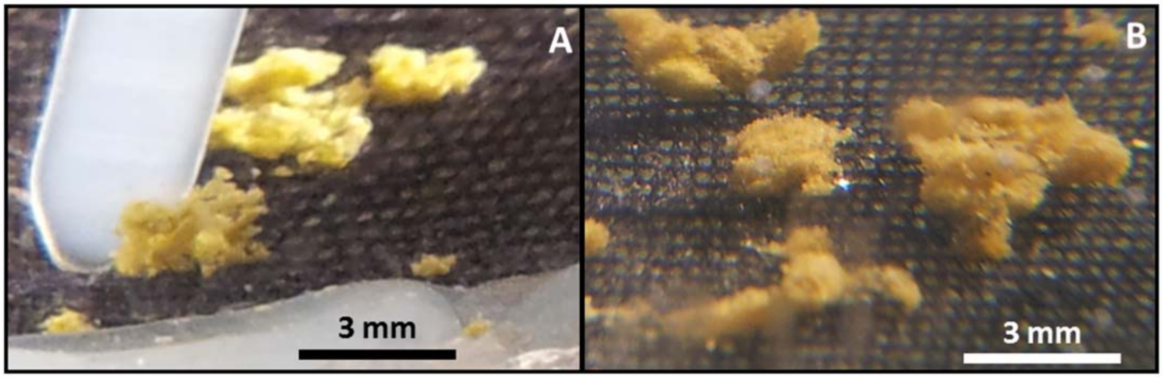

Particles formed during 5 days incubation in a flow-through roller tank (A) as compared to those formed in a closed system after an equal time period. The particles in the closed system were darker in color, faster sinking and stickier. The white plastic band is 3 mm in width and was used for size approximation during measurements.

Figure S4

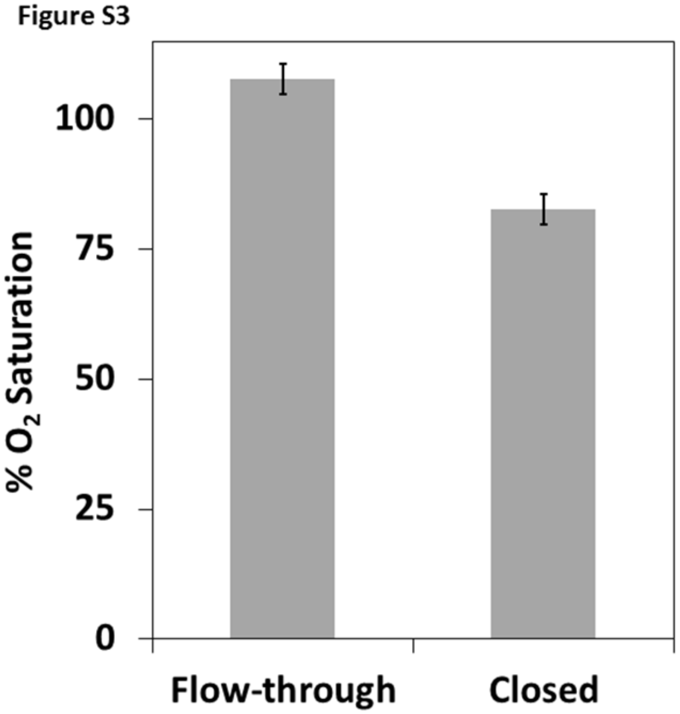

O_2 concentration as measured in water from the flow-through and closed roller tanks. The error bars represent technical replicates from 3 water samples taken from the outlets and 1 final measurement in the open roller tank. The flow-through roller tank was supersaturated, matching the ongoing diatom bloom in the lake at the time the experiment was held. The closed system was undersaturated suggesting respiration was dominating over O_2 produciton.
